# The Effects of Hematopoietic Growth Factors on Neurite Outgrowth

**DOI:** 10.1371/journal.pone.0075562

**Published:** 2013-10-08

**Authors:** Ye Su, Lili Cui, Chunshu Piao, Bin Li, Li-Ru Zhao

**Affiliations:** 1 Department of Neurology, Louisiana State University Health Sciences Center, Shreveport, Louisiana, United States of America; 2 Department of Neurosurgery, State University of New York Upstate Medical University, Syracuse, New York, United States of America; 3 Department of Cellular Biology and Anatomy, Louisiana State University Health Sciences Center, Shreveport, Louisiana, United States of America; University of Louisville, United States of America

## Abstract

Stem cell factor (SCF) and granulocyte colony-stimulating factor (G-CSF) are initially discovered as the essential hematopoietic growth factors regulating bone marrow stem cell proliferation and differentiation, and SCF in combination with G-CSF (SCF+G-CSF) has synergistic effects on bone marrow stem cell mobilization. In this study we have determined the effect of SCF and G-CSF on neurite outgrowth in rat cortical neurons. Using molecular and cellular biology and live cell imaging approaches, we have revealed that receptors for SCF and G-CSF are expressed on the growth core of cortical neurons, and that SCF+G-CSF synergistically enhances neurite extension through PI3K/AKT and NFκB signaling pathways. Moreover, SCF+G-CSF induces much greater NFκB activation, NFκB transcriptional binding and brain-derived neurotrophic factor (BDNF) production than SCF or G-CSF alone. In addition, we have also observed that BDNF, the target gene of NFκB, is required for SCF+G-CSF-induced neurite outgrowth. These data suggest that SCF+G-CSF has synergistic effects to promote neurite growth. This study provides new insights into the contribution of hematopoietic growth factors in neuronal plasticity.

## Introduction

Stem cell factor (SCF) and granulocyte colony-stimulating factor (G-CSF) were initially discovered as hematopoietic growth factors based on their effects to support the growth of hematopoietic stem cells or hematopoietic progenitor cells (HSCs/HPCs) [1,2]. C-kit, the receptor for SCF, and GCSFR, the receptor for G-CSF, are both expressed in HSCs/HPCs [3,4]. SCF and G-CSF are crucially involved in the proliferation, differentiation, and mobilization of HSCs/HPCs [5,6]. Convincing evidence has shown that SCF in combination with G-CSF (SCF+G-CSF) has synergistic effects on HSC/HPC mobilization [7].

Besides the primary effects of SCF and G-CSF in the hematopoietic system, accumulating evidence suggests that SCF and G-CSF also play roles in the central nervous system (CNS). SCF and G-CSF can pass through the blood-brain barrier [8,9] and have effects on neurogenesis and neuroprotection. It has been shown that receptors for SCF and G-CSF are also expressed in neural stem cells/neural progenitor cells (NSCs/NPCs) [8,10-12] and cerebral neurons [8,11]. SCF [10] and G-CSF [8] alone or in combination [12] promotes differentiation of NSCs/NPCs into neurons. In addition, systemic administration of SCF [11] and G-CSF alone [8,11] or in combination [11] in acute stroke reduces the infarction size and facilitates functional restoration.

Several lines of evidence support that SCF and G-CSF also play a role in neuronal plasticity. Mice that lack SCF [13] or ckit [14] display impaired long-term potentiation (LTP) and spatial learning and memory. G-CSF deficient mice also show cognitive impairment, LTP reduction, and poor neuronal networks in the hippocampus [15]. Moreover, our early study shows that treatment with SCF+G-CSF not SCF or G-CSF alone in chronic stroke induces a stable and long-term somatosensorimotor functional improvement [16], suggesting that neuronal network remodeling may be enhanced by SCF+G-CSF. Convincing evidence has shown that neuronal network rewiring is critically involved in functional recovery after stroke [17,18]. Using live brain imaging we have recently revealed that synaptogenesis and neuronal network formation in the peri-infarct cortex of chronic stroke brain are enhanced by SCF+G-CSF [19]. Stimulating neurite outgrowth and promoting new synapse formation are critical steps for building neuronal networks. The aim of the present study is to determine whether SCF and G-CSF have the effects on neurite outgrowth.

## Materials and Methods

All procedures have been approved by the Institutional Animal Care and Use Committee and are in accordance with the National Institutes of Health Guide for the Care and Use of Laboratory Animals.

### Hematopoietic growth factors

Recombinant rat SCF (PeproTech) and recombinant human G-CSF (Amgen) were used for this study. The concentration of SCF or G-CSF utilized in this study was 10ng/ml, unless otherwise noted.

### Cortical neuron culture

Cortical neurons were obtained from the embryonic brains at embryonic day 18 (E18) of Sprague–Dawley rats. Briefly, the cerebral cortex of the embryonic brains were dissected under an optical microscope (Zeiss Stemi DV4) and incubated in Hank’s Buffered Salt Solution (HBSS) containing papain (1.33 mg/ml) (Sigma) for 15 min at 37°C. The dispersed cerebral cortical tissues were then neutralized with serum-free neuronal culture medium containing DNAse I and trypsin inhibitor (Sigma) for 15 min at 37°C and dissociated into single cells. The dissociated neurons were grown in neuronal culture medium (Neurobasal medium, 2% B27 supplement and 0.5mM glutamine) (Life Technologies) in a humidified incubator at 37°C and 5% CO_2_. Fresh culture medium was replaced at 50% of the medium in each well every three days.

### Determination of neurite outgrowth

Neurite outgrowth was determined with three independent methods. 1) *To measure the length of neurites*. Cortical neurons were seeded onto the coverslips in 24-well plates that were pre-coated with poly-D-lysine (PDL) (10 µg/ml) (Sigma) at a density of 1x10^4^ cells/ml. Neurons were treated with medium alone or SCF and G-CSF for 24 h, fixed with 4% buffered-paraformaldehyde, and then processed for immunocytochemistry. The neurons (>100 neurons/coverslip) were then photographed with a Zeiss confocal microscope (Zeiss LSM 510 NLO) and the length of neurites was measured using Image-Pro Plus software 7.0 (Media Cybernetics). 2) *To use a Neurite Outgrowth Assay Kit* (*1µm*) (*Millipore*). The transwell inserts (Milicell inserts) contain a permeable membrane with 1µm pores at the base. The permeable membranes allow for projecting neurites to pass easily through the pores but not the cell bodies. The Milicell inserts were coated with collagen (10µg/ml) (BD Biosciences) and laminin (10µg/ml) (Millipore) for 24 h, respectively. Neurons were planted on the upper side of the membrane (1x10^5^ cells/well) for 48 to 96 h dependent on different experiments in this study. The neurons extended their neurites through the membrane to the underside of the insert membrane surface. By the end of experiment, inserts were removed from 24-well plates and the membranes were fixed in 100% methanol (-20°C). Membranes were then stained with Neurite Stain Solution, and cell bodies on the top of the membranes were removed by wiping with the flattened tip of a cotton swab. The neurites on the underside of the membrane were extracted with Neurite Stain Extraction Buffer. The Buffer was then transferred into a 96-well plate and the neurite extension was quantified on a spectrophotometer by reading absorbance at 562 nm. OD562 values were normalized to the Stain Extraction Buffer. 3) *To assess the neurites on transwell membranes with immunocytochemistry*. The procedures were similar to the method 2 stated above except using immunocytochemistry to visualize the neurites. The neurites on the underside of the insert membrane (≥ 5-6 fields/membrane) were captured with a Zeiss confocal microscope (Zeiss LSM 510 NLO) and analyzed with Image-Pro Plus software 7.0 (Media Cybernetics).

To block PI3k/AKT signaling, PI3K inhibitor, LY294002 (20µM) (Cell Signaling Technology), was added 60 min before SCF and G-CSF treatment. To block BDNF receptor, Trk B, a sheep anti-rat TrkB antibody (1:500) (catalog number OST00117W, Pierce Biotechnology) was added to neurons 60 min before exposure to SCF and G-CSF.

### Immunocytochemistry and histochemistry

Neurons were fixed with 4% buffered-paraformaldehyde for 15-20 min at room temperature or 100% (-20°C) methanol for 20 min. Nonspecific binding was blocked with 5% normal goat serum diluted by 1% bovine serum albumin (BSA) (IgG free) (Jackson ImmunoResearch Labs) and 0.25% Triton X-100 (Sigma) for 45 minutes at room temperature. Neurons were then incubated with primary antibodies overnight at 4°C. The primary antibodies used for this study included: mouse monoclonal anti-βIII tubulin (TuJ1) antibody (1:500) (catalog number T8578, Sigma), rabbit anti-c-kit (1:50) (catalog number SC-168, Santa Cruz Biotechnology) and rabbit anti-G-CSFR (1:50) (catalog number SC-694, Santa Cruz Biotechnology). Brain sections with omission of primary antibodies served as negative controls. Neurons were then incubated with secondary antibodies for 2 h at room temperature. The secondary antibodies used for this study were DyLight-549-conjugated goat anti-rabbit IgG (1:500) (catalog number 111-505-144, Jackson ImmunoResearch Labs), DyLight-488-conjugated goat anti-mouse IgG (1:500) (catalog number 115-485-166, Jackson ImmunoResearch Labs), and Cy2-conjugated goat anti-mouse (1:200) (catalog number 115-225-146, Jackson ImmunoResearch Labs). F-actin staining was performed by incubation of Alexa Fluor® 488 Phalloidin (1:200) (catalog number A12379, Life Technologies) for 1 h at room temperature. After washing with phosphate buffered saline (PBS), the neurons seeded on coverslips were mounted with ProLong Gold Antifade Reagent (Life Technologies), and the neurons grown in the culture wells were kept in PBS. A Zeiss confocal microscope (LSM 510 NLO) was used to obtain confocal images. The confocal images were acquired with a 60x oil lens for the neurons mounted on the coverslips or using a 40x water-immersion objective with 0.8 numerical aperture for the neurons kept in the cell culture wells.

### Western blotting

Proteins of cultured neurons were extracted with the M-PER Mammalian Protein Extraction Reagent and quantified with the Bicinchoninic Acid Protein Assay (Thermo Fisher Scientific). Proteins were loaded with 15% SDS-PAGE polyacrylamide gel and transferred to a PVDF membrane (Bio-Rad). Membranes were blocked with 5% non-fat milk and then incubated with primary antibodies overnight at 4°C. The primary antibodies used in this study were as follows: rabbit anti-phospho-AKT (1:1000) (catalog number 9271, Cell Signaling), rabbit anti-total AKT (1:1000) (catalog number 4691, Cell Signaling), rabbit anti-BDNF (1:200) (catalog number SC-20981, Santa Cruz Biotechnology), and rat anti-nerve growth factors (NGF) (1:200) (catalog number SC-32300, Santa Cruz Biotechnology). The membranes were then rinsed with Tris-buffered saline plus Tween (Hoefer) and incubated with secondary antibodies. The following secondary antibodies were used in this study: peroxidase-conjugated donkey anti-rabbit (1:2000) (catalog number 711-035-152, Jackson ImmunoResearch Labs), peroxidase-conjugated goat anti-rat (1:2000), (catalog number 112-035-003, Jackson ImmunoResearch Labs) and peroxidase-conjugated goat anti-mouse (1:2000) (catalog number 115-035-174, Jackson ImmunoResearch Labs). After triple washes, bands were visualized by an enhanced chemiluminescent substrate (Thermo, Fisher Scientific). A mouse anti- glyceraldehyde 3-phosphate dehydrogenase (GAPDH) (1:500) (catalog number G8795, Sigma) was used as an internal control. Western blot data were quantified by densitometric film scanning and normalized to GAPDH levels.

### RNA extractions, reverse transcription and quantitative real-time polymerase chain reaction

The total RNA from cultured primary neurons was extracted using the RNeasy Plus Mini Kit (Qiagen). The reverse transcription was performed using a High Capacity RNA-to-cDNA Kit (Applied Biosystems) with a total reaction volume of 20µl on a Mastercycler gradient PCR machine (Eppendorf). The cycling conditions were set at 37°C for 60 min, 95°C for 5 min, followed by 4°C until use. Quantitative real-time polymerase chain reaction (qRT-PCR) was carried out for all genes of interest in each sample using cDNA specific TaqMan Gene Expression Assays (Applied Biosystems) on an ABI Prism 7900HT Real-Time PCR System (Applied Biosystems). The probes/primers of the TaqMan Gene Expression Assays used for this study were rat BDNF (catalog number: 4453320, assay ID - Rn01484924_m1, Applied Biosystems) rat NGF (catalog number: 4453320, assay ID - Rn01533872_m1, Applied Biosystems) and rat beta-actin (ACTB) endogenous control (FAM™ Dye / MGB Probe, Non-Primer Limited) (catalog number: 4352933E, Applied Biosystems). Data analysis was based on the ΔΔCt method. In each 20 µl TaqMan reaction, cDNA was diluted in RNase free water to 9µl and was mixed with 1 µl TaqMan Gene Expression Assay and 10 µl TaqMan Universal PCR Master Mix (Applied Biosystems). This allowed for the consistent use of standardized thermal cycling conditions, 50°C for 2 min, 95°C for 10 min, followed by 40 cycles of 95°C for 15 sec and 60°C for 1 min. All qRT-PCRs were run in quadruplicate.

### Determination of phospho-IκBα

Cells were washed with ice-cold PBS. After being centrifuged at 4°C, the supernatant was discarded, and a volume of Complete Lysis Buffer equivalent was added. The mixture was then incubated on ice, and centrifuged at 4°C. The supernatant was collected, and the protein content was measured by a bicinchoninic acid protein assay. An ELISA kit was used to quantify phospho-inhibitory κB kinase α (IκBα) ^Ser32/Ser36^ (FunctionELISA^TM^ IκBα) (Active Motif) following SCF and G-CSF treatment.

### Nuclear extracts and nuclear factor κB binding activity assay

Nuclear extracts were harvested from cultured neurons using a Nuclear Extract Kit (Active Motif) by following the manufacturer’s protocol. Briefly, neurons were washed with ice-cold PBS containing phosphatase Inhibitors, collected and centrifuged at 4°C. The cell pellet was resuspended in Complete Lysis Buffer and incubated on ice on a rocking platform. After being centrifuged at 4°C, the supernatant was collected and stored at -80°C until use. An NFκB Transcription Factor Enzyme-linked Immunosorbent Assay (ELISA) Kit (p65) (Active Motif), which is designed for specific determination of activated nuclear factor κB (NFκB) in the rat tissue extracts, was used for the determination of NFκB transcriptional activity in rat cortical neurons according to manufacturer’s recommendations. To demonstrate binding specificity, a wild-type NFκB consensus oligonucleotide or mutant NFκB consensus oligonucleotide was added into the wells prior to adding the cell extract.

### Live neuron imaging

Primary cortical neurons were seeded at a concentration of 1x10^4^ cells/ml, 2.5ml/dish, in a 35mm-glass-bottom disk (Mat Tek Corporation), which was pre-coated with PDL (0.01%). Medium or SCF+G-CSF treatment was provided 8 h after seeding. NFκB inhibitor, BAY 11-7082 (10µM), was added 5 min prior to SCF+G-CSF treatment. A Leica SP5 confocal microscope equipped with CO_2_ and temperature control system was used for live neuron imaging. A 40x air objective lens was chosen to observe the cells in bright field mode. Live cell imaging was performed in an environment of 37°C by temperature probe feedback control and 5% CO_2_. Live cell images were recorded from 8h to 26 h after seeding with intervals of 30 min.

### Small interfering RNA transduction and gene silencing

One hour after adding small interfering RNAs (siRNAs), the neurons were treated with or without SCF and G-CSF. Silencer Select siRNA specific for NGF or BDNF, or siRNA negative control (Applied Biosystems) were delivered into neurons by binding with the Chimeric Rabies Virus Glycoprotein Fragment (RVG-9R) peptide (Bio-Synthesis). siRNAs at a concentration of 50 pmol/ml were mixed with RVG-9R at a concentration of 500 pmol/ml (siRNA vs. peptide in a 1:10 molar ratio), and the mixture was incubated for 10-15 min at room temperature in serum-free neuronal culture medium before use. Neurons were collected 36h or 72 h after exposure to siRNAs for determining gene expression or protein production, respectively.

### Statistical analysis

One- or two-way ANOVA followed by Bonferroni/Dunn correction were used to analyze the data collected from more than three groups. Student’s t-test was used for testing two-group data. Each experiment was repeated two or three times in an independent manner. Statistically significant difference between groups was determined as p-value is less than 0.05. Data are presented as mean ± standard error (SE).

## Results

### Receptors for SCF and G-CSF are expressed in the growth cones of neurites of cortical neurons

**Figure 1:**
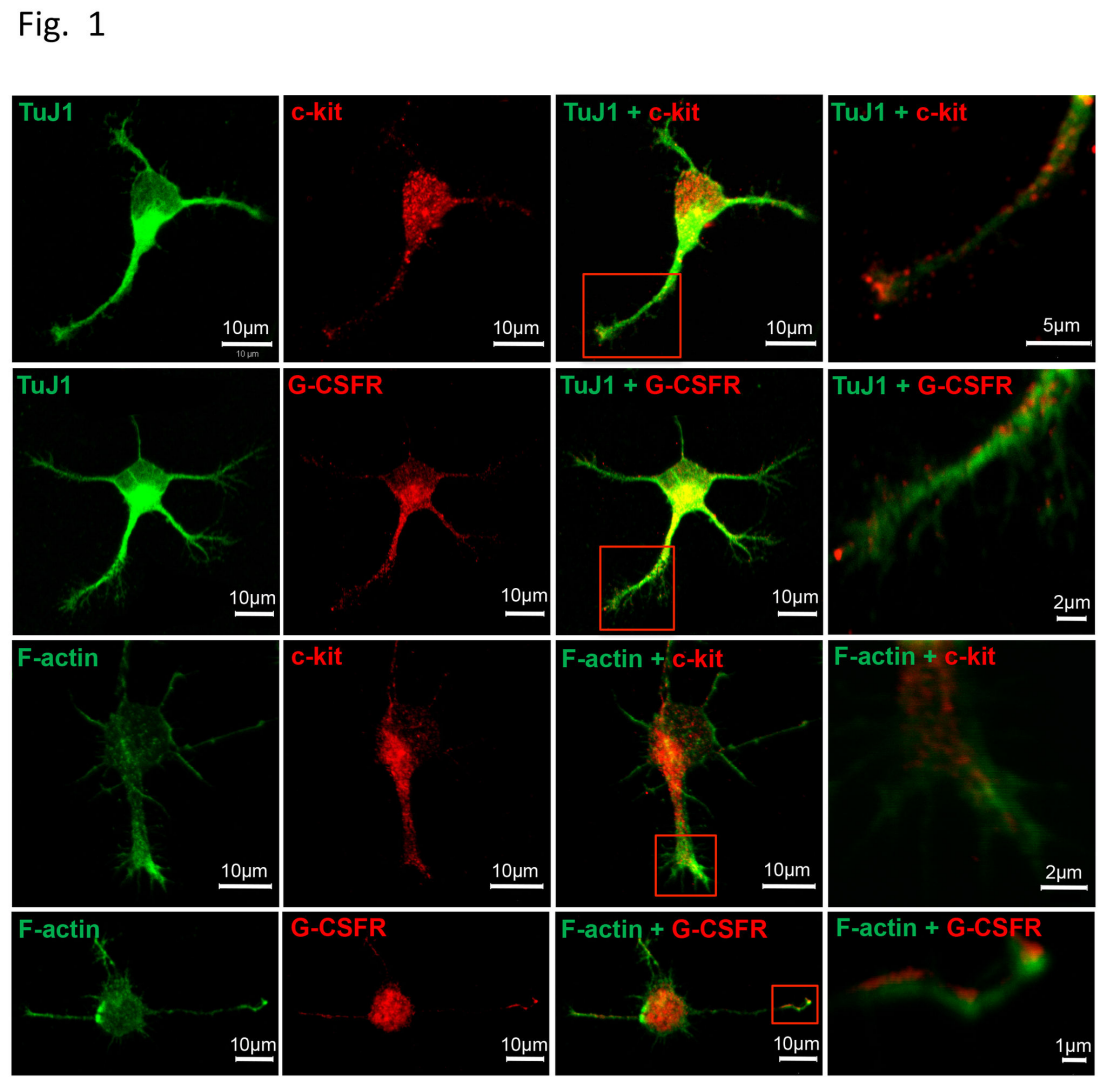
The receptors for SCF and G-CSF are expressed in primary cortical neurons and the growth cones. Cortical neurons were cultured for 24h and then processed for immunocytochemistry. c-kit (red), receptor for SCF. GCSFR (red), receptor for G-CSF. TuJ1 (green), the signature protein of neurons. F-actin (green), the marker of growth cones. The confocal images on the right columns are the high magnification images of the red boxes inserted in the adjacent panels. Note that c-kit and GCSFR are overlapped with TuJ1-positive neurons. Both c-kit and GCSFR are expressed in the neuron soma and neurites. Moreover, c-kit and GCSFR are also co-localized with F-actin in the neurite growth cones.

We first examined whether the receptors for SCF and G-CSF were expressed in the neurite growth cones of cortical neurons. Cortical neurons were cultured for 24 h, fixed and processed for immunocytochemistry. Confocal images showed that both c-kit (the receptor for SCF) and GCSFR (the receptor for G-CSF) were expressed on the neuronal soma and neuritis (Figure 1). This observation was consistent with previous findings that receptors for SCF and G-CSF were expressed in the cortical neurons [8,11]. Interestingly, we also found that c-kit and GCSFR were co-localized with a neurite growth cone marker, F-actin, (Figure 1) suggesting that hematopoietic growth factors, SCF and G-CSF, may have biological effects on neurons to regulate neurite outgrowth.

### Neurite outgrowth of cortical neurons is promoted by SCF+G-CSF

**Figure 2:**
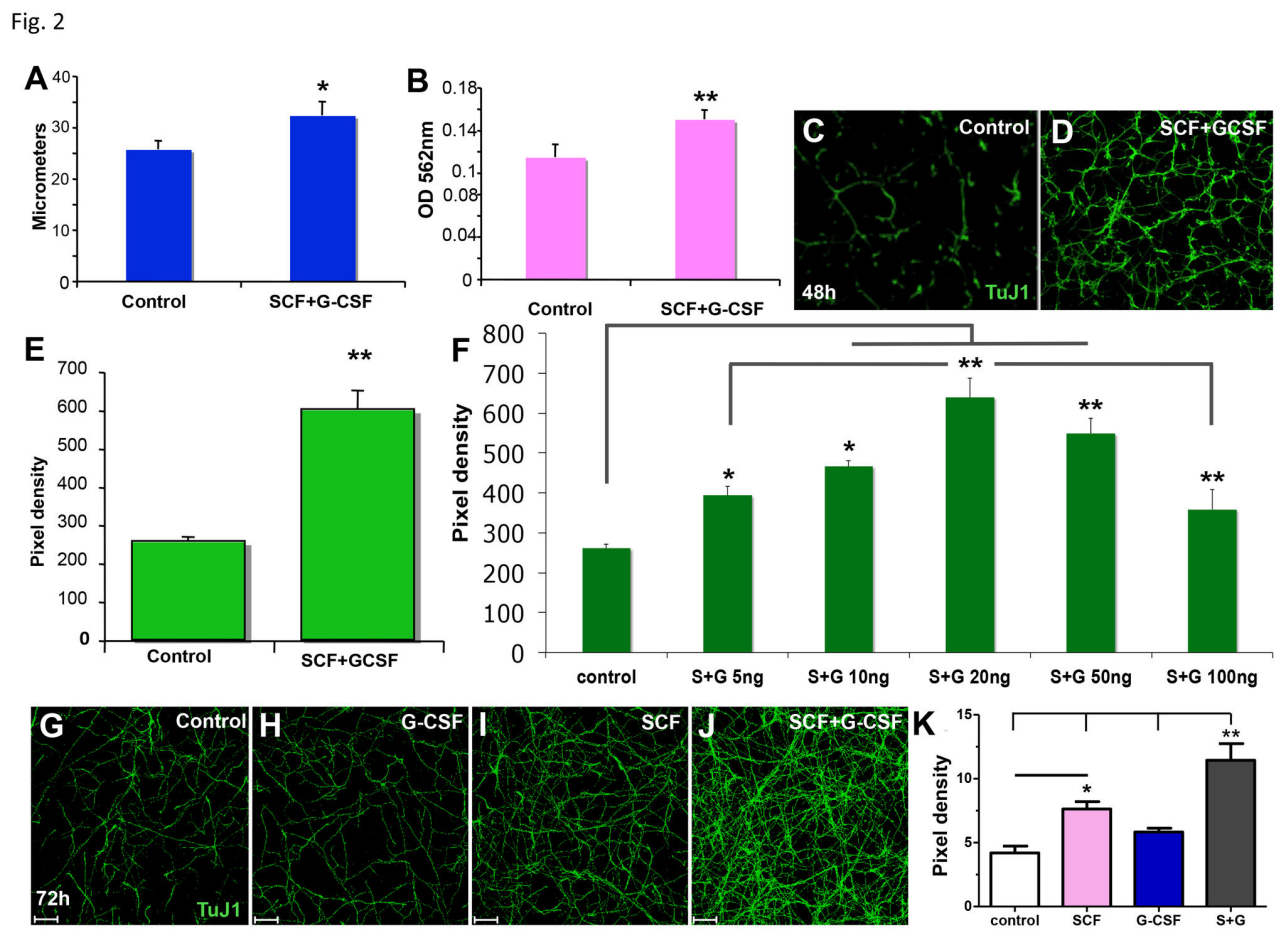
SCF+G-CSF promotes neurite outgrowth. (**A**) Measurements of the neurite length 24h after seeding. Note that the neurite length is significantly increased by SCF+G-CSF. (**B**) SCF+G-CSF promotes neurite extension 48h after plating. Neurite outgrowth was determined with a Neurite Outgrowth Quantification Assay Kit. (**C**-**E**) Neurite extension and neuronal network density are enhanced by SCF+G-CSF. Cortical neurons were cultured on transwell membranes with or without SCF+G-CSF treatment for 48h. Neurite outgrowth and neurite network density were examined with immunofluorescent staining on the underside membranes of transwells. (**C** and **D**) Immunofluorescent images. (**E**) Quantification of neurite extension and neuronal network density. (**F**) Dose-response data. Note that SCF+G-CSF induces neurite outgrowth in a dose-dependent manner. (**G**-**K**) Synergy assay data. Cortical neurons were plated on transwell membranes with medium alone (control), G-CSF, SCF or SCF+G-CSF treatment (20ng/ml) for 72h. (**G**-**J**) Confocal images of the neurites on the underside membranes of transwells by immunocytochemical staining. (**K**) Quantification data of synergy assay. Note that SCF alone or in combination with G-CSF (SCF+G-CSF) significantly increases neurite extension, and that SCF+G-CSF shows synergistic effects on the enhancement of neurite outgrowth. *p<0.05, **p<0.01. Mean ± SE; n=3-4. SCF or G-CSF, 20ng/ml unless otherwise noted.

Next we wanted to determine whether SCF and G-CSF have a direct effect on cortical neurons to promote neurite outgrowth and neuronal network formation. Three independent methods were used to examine the effects of SCF and G-CSF on neurite outgrowth or neuronal network formation in the primary cortical neuronal cultures. First, neurite outgrowth was determined by measuring neurite length. The cortical neurons were treated with or without SCF+G-CSF for 24 h, and the treatment started when seeding the neurons. We observed that SCF+G-CSF treatment resulted in a significant increase in the length of neurites (Figure 2A). The second method was the determination of neurite outgrowth using a Neurite Outgrowth Assay Kit. The cortical neurons were cultured for 48h in transwells and treated with or without SCF+G-CSF when seeding the neurons in the transwells. We found that SCF+G-CSF treatment showed a significant increase in neurite outgrowth (Figure 2B). To further confirm these findings, we utilized a third method, which was to determine the neurite extension by staining the neurites on transwell membranes. In this experiment, the cortical neurons were grown in the transwells for 48h (Figure 2 C-E) or 72h (Figure 2 G-K), and neuron culture medium (control), SCF, G-CSF, or SCF+G-CSF was added into the transwells at the time of plating. We found that SCF+G-CSF induced a significant enhancement of neurite outgrowth and neuronal network formation (Figure 2C-E). Moreover, in a dose dependent assay, we observed that the effective doses of SCF+G-CSF to promote neurite extension ranged from 10ng/ml to 50ng/ml and that the maximal effective dose was 20ng/ml (Figure 2F). The experiment for the dose dependent assay was performed in the same manner as stated in the third method for neurite outgrowth assay where cortical neurons were plated into the transwells for 48h. In addition, using the highest effective dose (20ng/ml), we found that SCF alone or SCF+G-CSF induced significant enhancement of neurite outgrowth, whereas G-CSF alone only showed a trend toward increased neurite extension but did not reach a significant level (Figure 2G-K). Interestingly, SCF+G-CSF-induced neurite outgrowth was much greater than SCF or G-CSF alone (Figure 2G-K). Similar results were seen with a dose of 10ng/ml (data not shown). These data suggest that hematopoietic growth factors, SCF+G-CSF, have direct and synergistic effects on the regulation of neurite outgrowth of cortical neurons.

### PI3K/AKT signaling is required for SCF+G-CSF-induced neurite outgrowth

**Figure 3:**
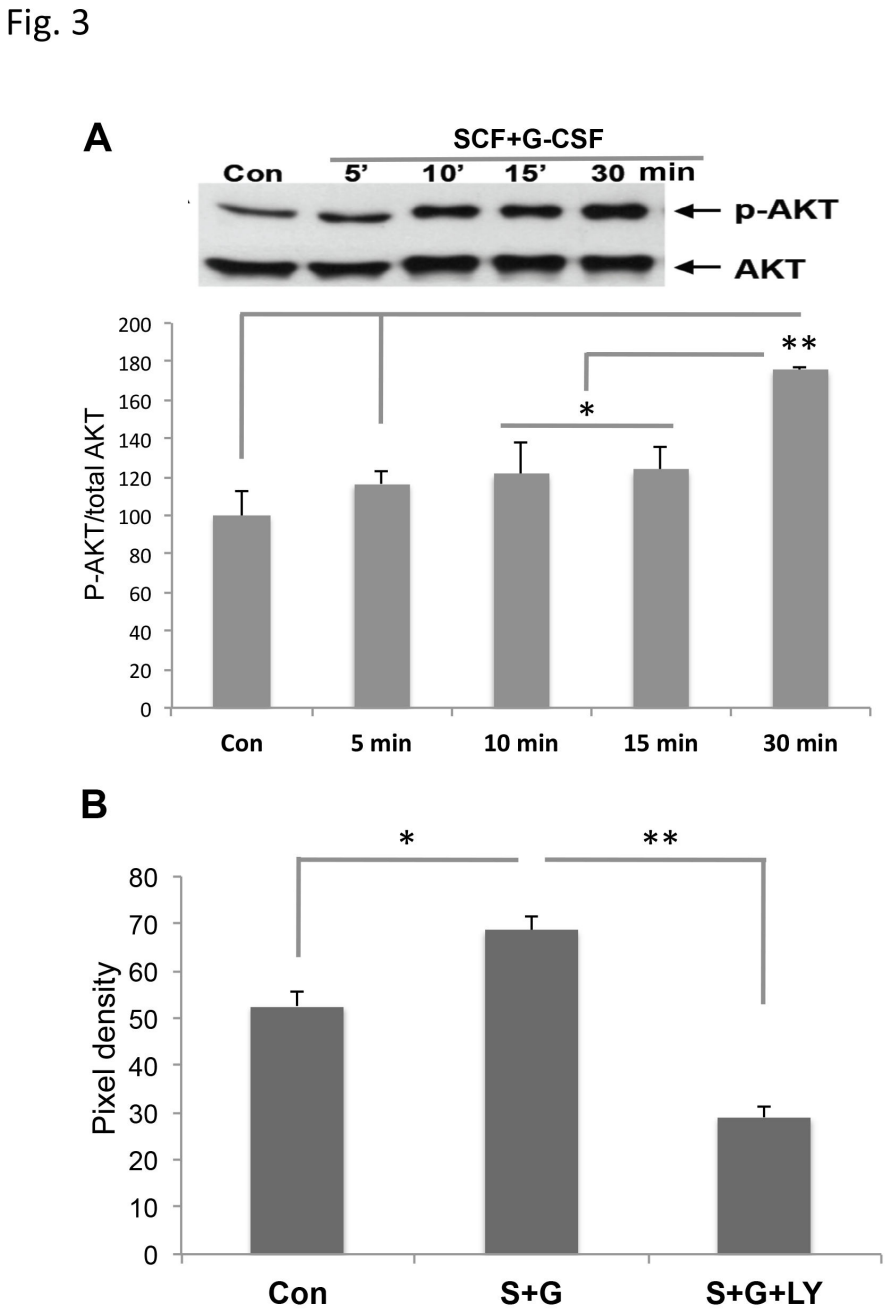
The involvement of PI3K/AKT signaling in SCF+G-CSF-induced neurite outgrowth. (**A**) Western Blotting data. Upper panel (bands): representative bands of phosphorylated AKT (p-AKT) and total AKT (AKT). Lower panel (bar graph): semi-quantitative of the phosphorylated AKT. Note that SCF+G-CSF causes phosphorylation of AKT in a time-dependent manner. Cortical neurons were cultured for 24h and treated with or without SCF+G-CSF, and protein extracts of neurons were collected at selected time to determine phosphorylated AKT. (**B**) SCF+G-CSF-induced neurite outgrowth is blocked by PI3K signaling inhibitor, LY294002 (LY). Cortical neurons were seeded onto the membranes of transwells for 24h and treated with medium alone (Control), SCF+G-CSF, or SCF+G-CSF+ LY294002 for two days before immunocytochemistry. PI3K inhibitor, LY294002 (20µm), was added 1h prior to SCF+G-CSF treatment. Con: control medium, S+G: SCF+G-CSF, S+G+LY: SCF+G-CSF+LY294002. *p<0.05, **p<0.001. Mean ± SE. N=3.

Next we sought to determine how SCF+G-CSF promotes neurite outgrowth. AKT is a direct downstream target molecule of PI3K. Previous studies have shown that the PI3K/AKT signal transduction pathway is involved in neurite outgrowth [20,21]. Therefore, we hypothesized that SCF+G-CSF-induced neurite extension is regulated through the PI3K/AKT signal transduction pathway. To test this hypothesis, we performed the following experiment. Cortical neurons were cultured for 24h and then exposed to medium alone or SCF+G-CSF. Western Blotting was utilized to examine phosphorylation of AKT 5, 10, 15, and 30 min after SCF+G-CSF treatment. We found that SCF+G-CSF caused AKT phosphorylation in a time-dependent manner and that the highest level of phosphorylated AKT was seen 30 min after exposure to SCF+G-CSF (Figure 3A). In addition, we also observed that pre-treatment with a PI3K inhibitor, LY294002 (20µM), significantly prevented SCF+G-CSF-induced neurite outgrowth (Figure 3B) (Control vs. SCF+G-CSF, 52.59 ± 3.23 vs. 68.73 ± 2.99, p <0,05; SCF+G-CSF vs. SCF+G-CSF+LY, 68.73 ± 2.99 vs. 29.02± 2.33, p <0.01). These data indicate that PI3K/AKT signaling is involved in SCF+G-CSF-induced neurite extension.

### SCF+G-CSF promotes neurite extension through the regulation of nuclear factor-kappa B

We then wanted to determine the role of nuclear factor-kappa B (NFκB) in SCF+G-CSF-mediated neurite outgrowth. NFκB is activated by PI3K/AKT signaling [22]. NFκB is the family of transcription factors that critically participate in the regulation of a variety of gene expressions that control cell survival, proliferation and differentiation, and neurite growth [23]. NFκB consists of five structurally related proteins: p50, p52, p65 (Rel-A), Rel-B and Rel-C. The p50/p65 heterodimer is the most abundant and ubiquitously expressed NFκB form [24] and is also the primary active form of NFκB in the CNS [23]. It has been demonstrated that the p65 subunit of the p50/p65 heterodimer is the functional regulator of NFκB and a strong activator for gene expression [25,26]. NFκB interacts with the IkappaB (IκB) family of inhibitory proteins to maintain an inactive complex in the cytosolic compartment. IκBα is the most commonly expressed inhibitor of NFκB. Phosphorylation of IκBα results in the translocation of NFκB heterodimer into the nucleus to bind DNA at Kappa-B binding motifs and promote gene expression [24].

**Figure 4:**
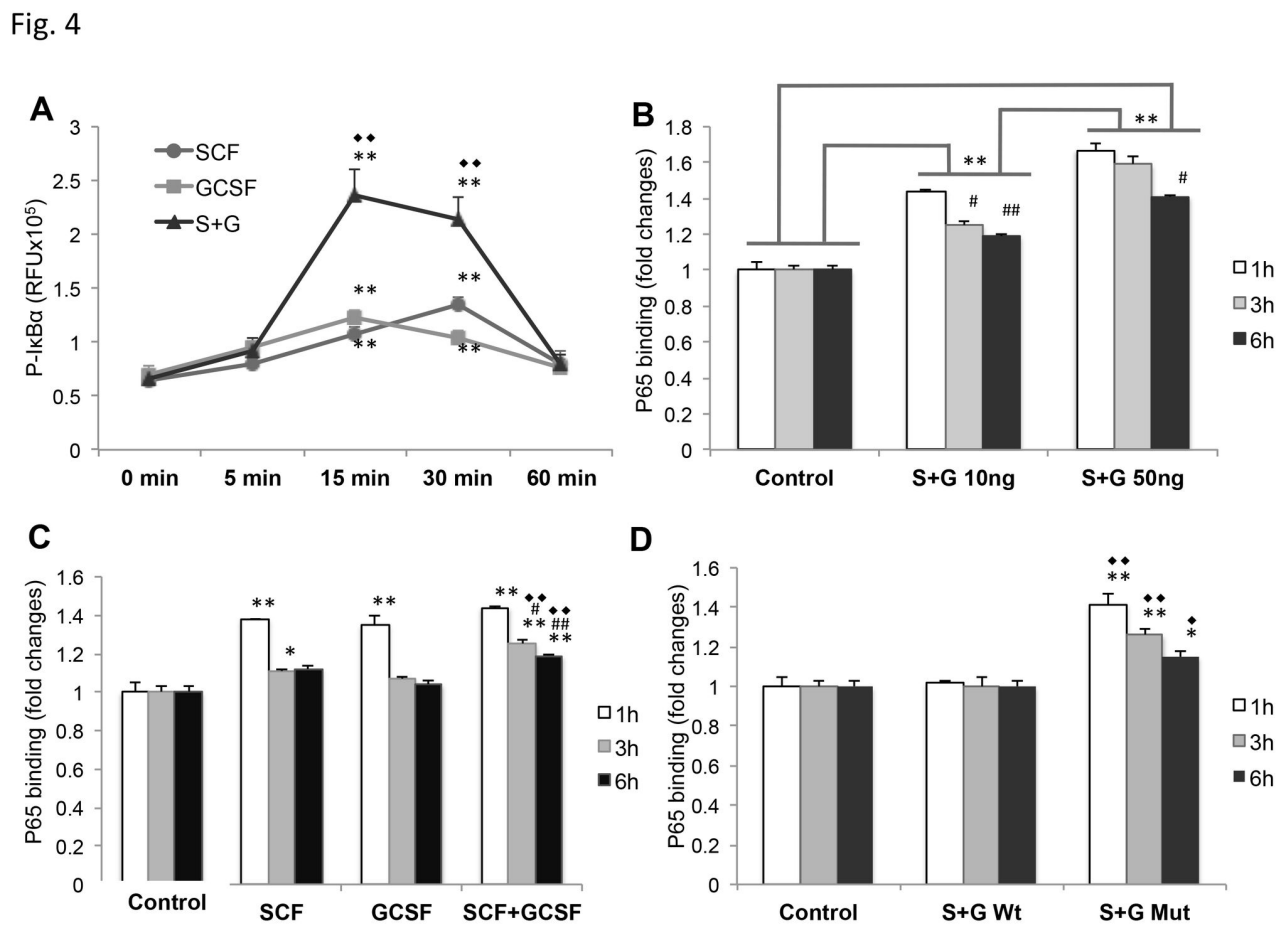
NFκB activation and transcriptional activity are enhanced by SCF+G-CSF. (**A**) NFκB activation assay data. NFκB activation was determined by IκBα phosphorylation with a FunctionELISA IκBα kit for the quantification of phosphorylated IκBα. Note that SCF and G-CSF alone or in combination induces a significant elevation of phosphorylated IκBα in a time-dependent manner. Markedly, the level of phosphorylated IκBα is significantly increased in SCF+G-CSF-treated neurons at 15 and 30 min after treatment as compared to SCF or G-CSF alone treatment. **p < 0.01: 0 min vs. 15 min, 0 min vs. 30min. ♦♦p < 0.01: SCF+G-CSF vs. SCF, SCF+G-CSF vs. G-CSF at 15 and 30 min after treatment. Mean ± SE, n=6. (**B**) Dose dependent assay of NFκB transcriptional activity. Quantification of NFκB binding activity was performed using a NFκB Transcription Factor ELISA Kit (p65). Note that both 10 ng/ml of SCF+G-CSF and 50 ng/ml of SCF+G-CSF enhance NFκB binding 1, 3 and 6 h after treatment in comparison with medium control (** p < 0.01). In addition, NFκB binding activity in the treatment of 50 ng/ml of SCF+G-CSF is greater than those of 10 ng/ml of SCF+G-CSF at 1, 3 and 6 h after treatment (** p < 0.01). In the dose of 10 ng/ml, NFκB binding activity is significantly increased 1h after treatment as compared to 3h (# p < 0.05) or 6h (# # p < 0.01) post-treatment. In the dose of 50 ng/ml, a significantly greater NFκB binding activity is seen 1h after treatment than 6h post-treatment (# p < 0.05). Mean ± SE, n=3. (**C**) NFκB binding activity is changed by SCF and G-CSF alone or in combination treatment. Note that SCF, G-CSF, and SCF+G-CSF cause significantly increases in NFκB binding activity 1h after treatment as compared to the controls (** p < 0.01). However, only SCF+G-CSF shows a long-lasting enhancement of NFκB binding activity as this treatment induces a significant elevation of NFκB binding activity 3 and 6 h after treatment when compared to SCF or G-CSF alone treatment (♦♦p < 0.01). SCF also displays a higher level of activated NFκB 3h post-treatment than the controls (* p < 0.05), whereas there is no difference between SCF and the controls at 6h after treatment. Mean ± SE, n=3. (**D**) NFκB specific binding assay data. Note that SCF+G-CSF-induced the long-lasting enhancement of NFκB binding activity is completely blocked by wild-type (Wt) NFκB oligonucleotide, whereas a mutant NFκB oligonucleotide (Mut NFκB) fails to prevent the SCF+G-CSF-induced the long-lasting enhancement of NFκB binding activity. Wt: wild-type NFκB oligonucleotide. Mut: mutant NFκB oligonucleotide. ** p < 0.01, * p < 0.05, control vs. SCF+G-CSF+ Mut NFκB. ♦♦ p < 0.01, ♦ p < 0.05, SCF+G-CSF+ Wt NFκB vs. SCF+G-CSF+ Mut NFκB. Mean ± SE, n=3.

Based on the previous findings mentioned above, we then sought to determine the effects of SCF+G-CSF on NFκB functioning through two steps: 1) to examine whether SCF+G-CSF can induce IκBα phosphorylation (NFκB activation assay), and 2) to identify whether SCF+G-CSF can enhance NFκB binding activity by determination of p65 binding in the nuclei (NFκB transcriptional activity assay). To obtain the evidence that SCF+G-CSF can induce IκBα phosphorylation, cortical neurons were plated for 24 h and treated with medium alone, SCF, G-CSF, or SCF+G-CSF, and proteins were then extracted from the neurons at 0 (medium alone), 5, 15, 30, and 60 min. The levels of phosphorylated IκBα were quantified with a Function ELISA IκBα kit, which is specific and sensitive for the phosphorylated form of IκBα. We observed that SCF and G-CSF alone or in combination led to IκBα phosphorylation in a time-dependent manner. SCF, G-SCF or SCF+G-CSF all caused significant increases of IκBα phosphorylation at 15 and 30 min after treatment as compared to 0 min (p <0.001) (Figure 4A). Interestingly, the level of SCF+G-CSF-induced IκBα phosphorylation was much greater than SCF or G-CSF alone at 15 or 30 min (p <0.001) (Figure 4A). At 15 min post-treatment, SCF+G-CSF caused a 2.2-fold increase in the phosphorylated form of IκBα as compared to SCF, and a 1.9-fold increase as compared with G-CSF. At the 30 min post-treatment, SCF+G-CSF displayed a 1.6-fold increase in IκBα phosphorylation when compared to SCF and a 2-fold increase in comparison with G-CSF. These data suggest that SCF and G-CSF have the capability to trigger the phosphorylation of IκBα and that SCF+G-CSF has synergistic effects on IκBα phosphorylation.

Next we sought to determine the effects of SCF and G-CSF on NFκB transcriptional binding by examining the activated p65 in the nuclei. To assess the activated NFκB in the nuclei, cortical neurons were cultured for 24h and then treated with medium, SCF, G-CSF, or SCF+G-CSF at 10 ng/ml or SCF+G-CSF at 50 ng/ml. The nuclei of cortical neurons were extracted at 1, 3, and 6 h after treatment, and NFκB transcriptional binding was examined with an NFκB Transcription Factor ELISA Kit (p65), which is designed specifically for the determination of activated NFκB in rat tissue extracts. We observed that SCF and G-CSF facilitated NFκB transcriptional binding in a dose-dependent, time-dependent, combination-dependent, and binding site-dependent manner. SCF+G-CSF in the dose of 10 ng/ml or 50 ng/ml caused a significant increase in NFκB binding at 1, 3, and 6 h after treatment as compared to the medium control (p < 0.01) (Figure 4B). In addition, 50 ng/ml of SCF+G-CSF induced a much higher NFκB binding than those treated with 10 ng/ml of SCF+G-CSF 1, 3, and 6 h after treatment (p < 0.01) (Figure 4B). In both 10 ng/ml of SCF+G-CSF and 50 ng/ml of SCF+G-CSF treatment, NFκB binding activity at 1h post-treatment was greater than 3h (p < 0.05, 1h vs. 3h in 10 ng/ml of SCF+G-CSF) and 6h (p < 0.01, 1h vs. 6h in 10 ng/ml of SCF+G-CSF) (p < 0.05, 1h vs. 6h in 50 ng/ml of SCF+G-CSF) after treatment (Figure 4B). When we assessed the effects of SCF and G-CSF alone or in combination at a dose of 10 ng/ml on NFκB binding, we found that SCF, G-CSF, or SCF+G-CSF all enhanced NFκB binding 1h after treatment as compared to the controls (p <0.01) (Figure 4C). However, only SCF+G-CSF caused long-lasting enhancement of NFκB transcriptional binding because the NFκB binding activity was significantly increased in 1, 3, and 6 h after treatment in comparison with the medium controls at 1, 3, and 6 h post-treatment (p <0.01) (Figure 4C) or as compared to SCF or G-CSF alone at 3 and 6 h post-treatment (p <0.01) (Figure 4C). The SCF alone treatment also showed a significant increase in NFκB binding 3h after treatment when compared to the controls (p < 0.05), but the difference between SCF and the medium control was not seen at 6 h (Figure 4C). Moreover, we also observed that the SCF+G-CSF-induced long-lasting enhancement of NFκBp65 binding activity was dependent on the Kappa-B binding sequence on DNA. When adding a 20-fold excess of an NFκBp65 wild-type (WT) consensus oligonucleotide containing the κB consensus binding sequence as a competitor to block the specific binding site of NFκB, the SCF+G-CSF-induced long-lasting enhancement of NFκB binding activity was completely prevented (control vs. SCF+G-CSF+WT NFκB, p > 0.05) (Figure 4D). In contrast, when adding a mutant consensus NFκBp65 oligonucleotide (Mut NFκB), which has no effects to bind the Kappa-B binding sequence on DNA, SCF+G-CSF-induced the long-lasting enhancement of NFκB binding activity was not affected (1h and 3h: p < 0.01, SCF+G-CSF+Mut NFκB vs. control or SCF+G-CSF+WT NFκB) (6h: p < 0.05, SCF+G-CSF+Mut NFκB vs. control or SCF+G-CSF+WT NFκB) (Figure 4D).

**Figure 5:**
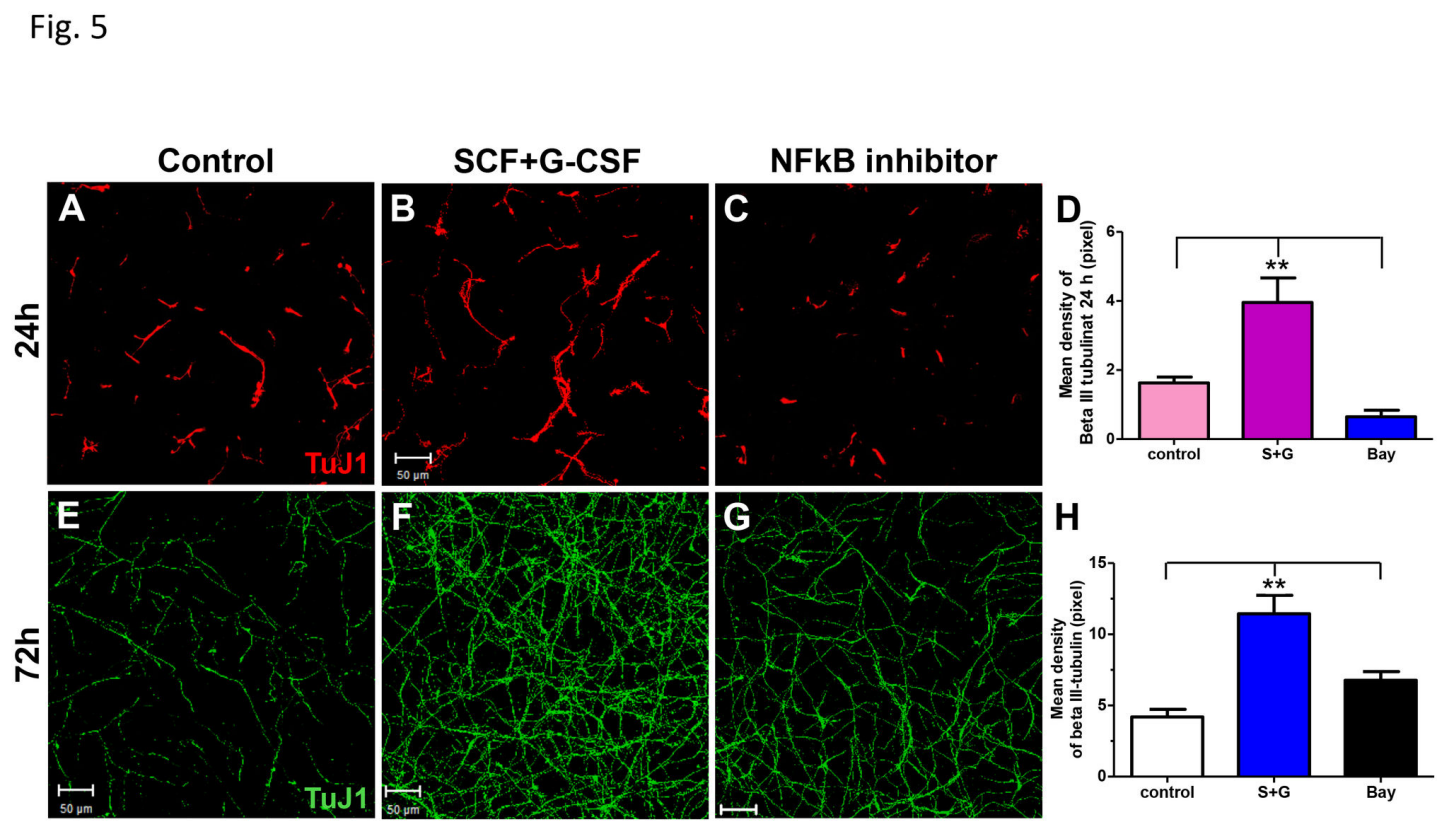
SCF+G-CSF-induced neurite outgrowth is regulated by NFkB. (**A**-**D**) Neurite outgrowth was examined 24h after seeding. (**E**-**H**) Neurite outgrowth was determined 72h after seeding. Cortical neurons were cultured for 2h, and NFkB inhibitor, Bay 11-7082 (Bay) (10µM for **A-D**, 5µM for **E-H**), was then added 5min (**A**-**D**) or 60min (**E**-**H**) before SCF+G-CSF treatment. (**A**-**C**, **E**-**G**) Representative confocal images of the neurites on the underside of transwell membranes. (**D** and **H**) Bar graphs represent quantification of neurite outgrowth with different treatment. Note that the SCF+G-CSF-induced neurite extension is significantly prevented by the NFkB inhibitor. Scale bars, 50µm. ** p < 0.01, Mean ± SE, n=4.

**Figure 6:**
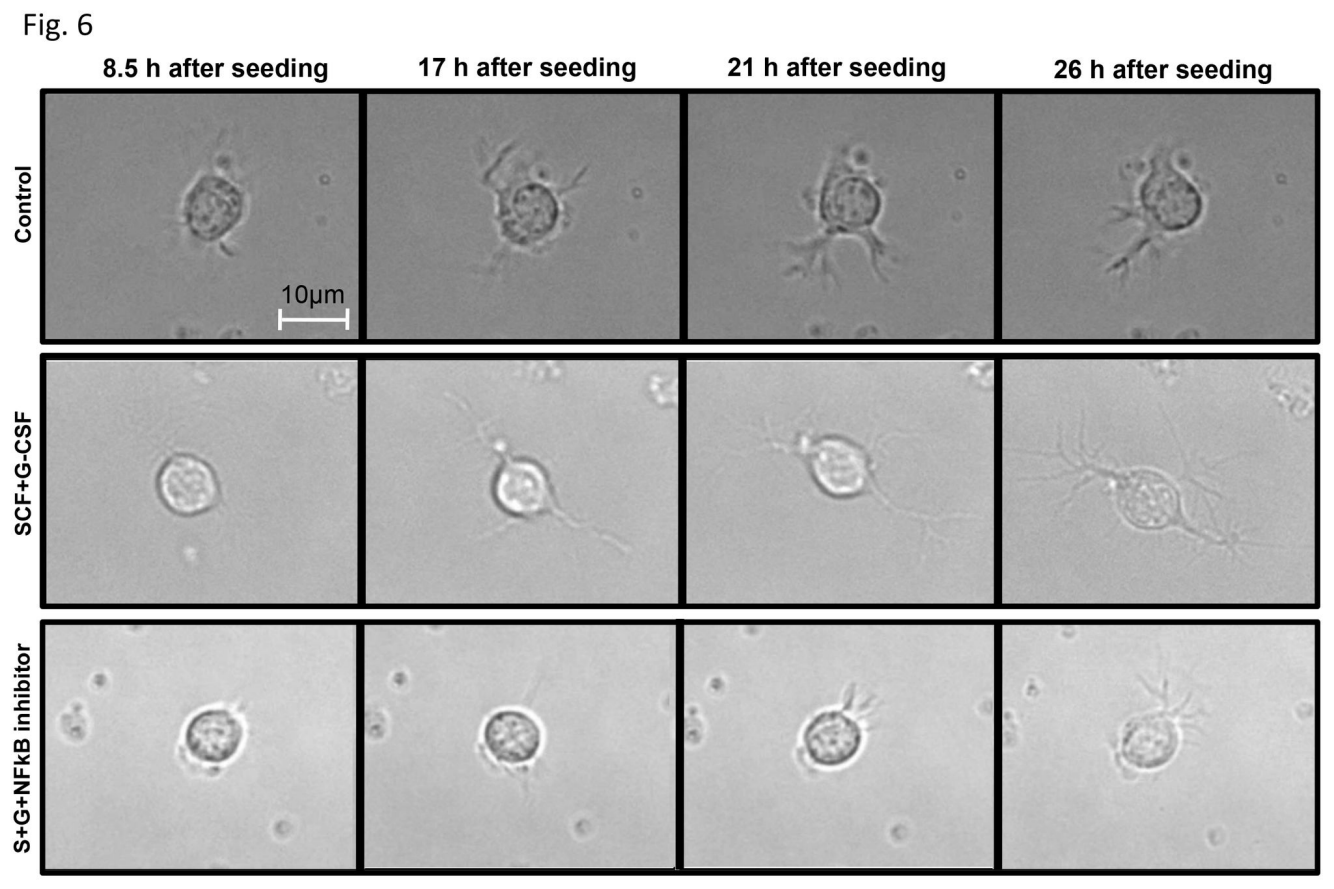
Live neuron images for neurite outgrowth. Note that SCF+G-CSF treatment leads to a marked increase in neurite extension, and that NFκB inhibitor, BAY 11-7082, prevents the SCF+G-CSF-induced the enhancement of neurite outgrowth. The images were selected at different time points from a movie recording neurite outgrowth during 8-26h after seeding (see Movies S1, S2, and S3).

Next we examined whether NFκB is a key player in the SCF+G-CSF-induced enhancement of neurite outgrowth. NFκB inhibitor (BAY 11-7082) was then added to neurons 1h before SCF+G-CSF treatment and 2h after seeding, and neurite outgrowth was determined 24h (Figure 5A-D) or 72h (Figure 5E-H) after treatment. We observed that SCF+G-CSF-induced enhancement of neurite outgrowth was significantly prevented by the NFκB inhibitor (Figure 5). In addition, we also used a live cell imaging approach to further confirm the role of NFκB in the SCF+G-CSF-induced enhancement of neurite outgrowth. Live neuron imaging was recorded at 30 min-intervals during 8-26 h after seeding. We chose the period of 8-26h post-planting neurons for imaging the neurite outgrowth because it has been shown that rapid neurite outgrowth occurs during this period [27]. Cortical neurons were treated with medium alone, SCF+ G-CSF, or SCF+ G-CSF +NFκB inhibitor. The NFκB inhibitor was added 1h before SCF+ G-CSF treatment, and the neurons were exposed to the treatment at 8h after plating. We observed that SCF+G-CSF treatment showed a dramatic increase in neurite extension during 8-26h after seeding. Compared to medium alone, SCF+G-CSF induced a 1.99-, 1.82-, or 1.88-fold increase in neurite length at 17, 21 or 26h after planting, respectively. In addition, a 1.2-, 1.67-, or 2.67-fold increase in neurite branching by SCF+G-CSF was seen at 17, 21 or 26h after planting, respectively, when compared to the medium control. Moreover, SCF+G-CSF -induced neurite extension was completely blocked by BAY 11-7082 (Figure 6). Time-lapse movies (Movie S1, Movie S2 and Movie S3) showed that the direction of neurite outgrowth was changed dynamically over time and that neurite extension and retraction also occurred together during the recording period. When compared to the medium control or SCF+G-CSF +NFκB inhibitor, SCF+G-CSF-treated neuron displayed polarization and neurite outgrowth at a much faster rate after treatment. Our observation suggests that NFκB is required for SCF+G-CSF-induced enhancement of neurite outgrowth.

### Brain-derived neurotrophic factor is the mediator for SCF+G-CSF-induced neurite outgrowth

**Figure 7:**
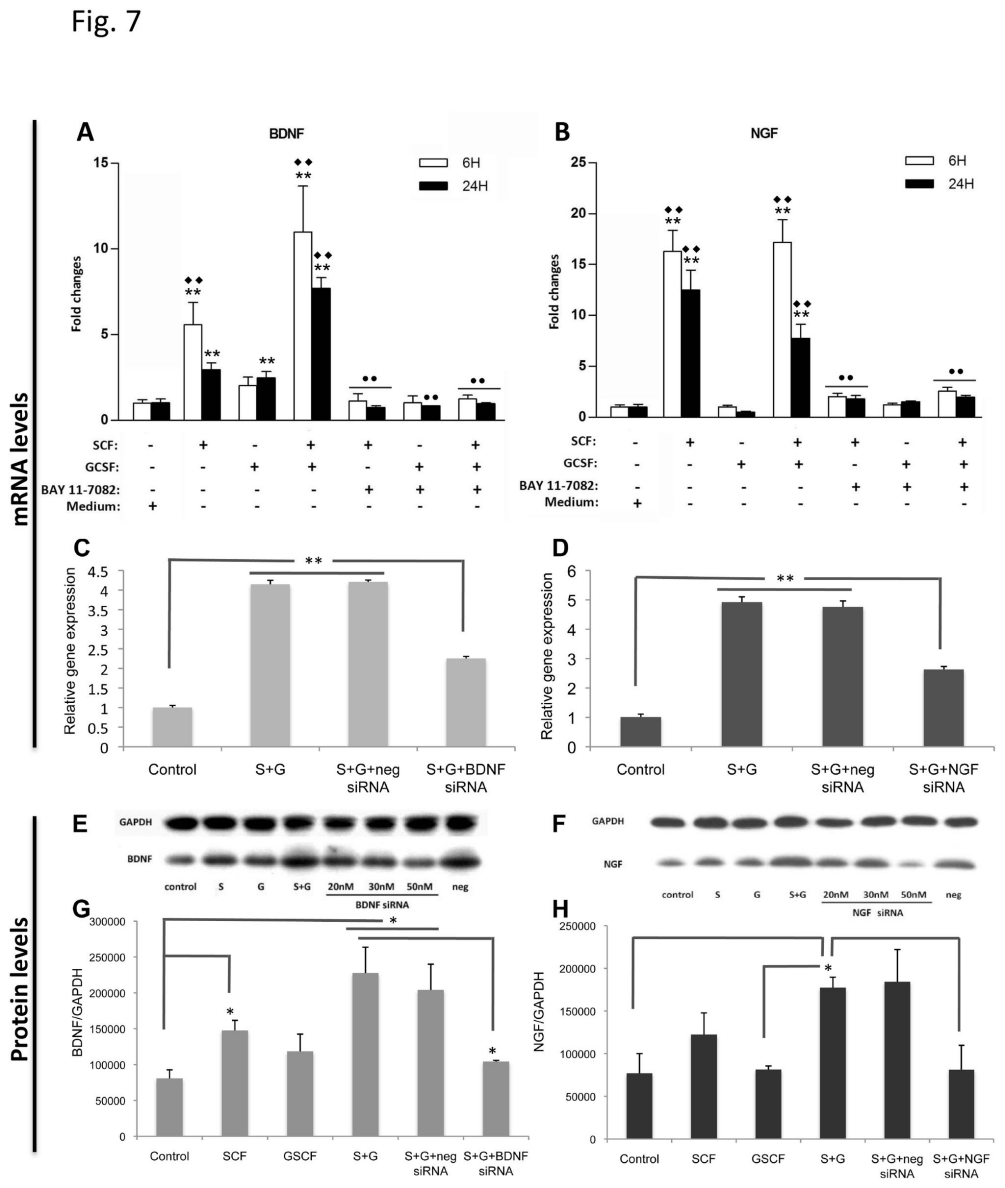
SCF+G-CSF upregulates BDNF and NGF at both the levels of transcription (mRNA) and translation (protein). (**A** and **B**) BDNF and NGF gene expression after SCF, G-CSF, or SCF+G-CSF treatment. Quantification of BDNF and NGF gene expression was performed with Real-Time PCR. (**A**) Quantification data for BDNF gene expression. Note that SCF or SCF+G-CSF induces a significant increase in BDNF gene expression at both 6 and 24h after treatment as compared to medium controls (**p < 0.01). The levels of BDNF gene expression are much higher in SCF+G-CSF-treated neurons than those in SCF or G-CSF-treated at both 6 and 24h after treatment (◆◆p<0.01). SCF also causes a significantly higher level of BDNF gene expression than those of G-CSF at 6 h post-treatment (◆◆p<0.01). G-CSF only induces an increase in BDNF gene expression at 24h (**p < 0.01) but not at 6h post-treatment as compared to medium controls. SCF-, G-CSF- (24h only), or SCF+G-CSF-induced upregulation of BDNF gene expression is significantly blocked by BAY 11-7082 (●● p < 0.01). (**B**) Quantification data for NGF gene expression. Note that both SCF and SCF+G-CSF treatments result in a significant upregulation of NGF gene expression 6 and 24 h post-treatment when compared to medium controls (** P < 0.01) or G-CSF (◆◆p<0.01). BAY 11-7082 significantly prevents SCF- or SCF+G-CSF-induced upregulation of NGF gene expression (●● p < 0.01). Mean ± SD, n=3-4. (**C** and **D**) SCF+G-CSF-induced the upregulation of BDNF and NGF gene expression is prevented by siRNAs against BDNF (**C**) or NGF (**D**). Gene expression was examined 36h after adding siRNAs. Neg: negative controls for siRNA. **p < 0.01. Mean ± SE. N=3-4. (**E**-**H**) SCF+G-CSF-induced increases of BDNF and NGF protein production are inhibited by siRNAs against BDNF (**E**, **G**) or NGF (**F**,**H**) 72h after adding siRNAs to neurons. (**E**, **F**) Representative Western-Blot bands for BDNF (**E**) and NGF (**F**) proteins after different treatments. S: SCF. G: G-CSF. S+G: SCF+G-CSF. Neg: negative controls for siRNAs. (**G** and **H**) Semi-quantification of Western-blot data for BDNF (**G**) and NGF (**H**) proteins. *p < 0.05. Mean ± SE, n=3-4.

Next we sought to identify the SCF+G-CSF/NFκB-promoted function genes that are critically involved in the SCF+G-CSF-induced enhancement of neurite outgrowth. We targeted the neurotrophic factors, BDNF and NGF, because previous studies have shown that BDNF and NGF are essential to neurite outgrowth [28,29]. In addition, it has been shown that both BDNF and NGF gene expression are promoted by NFκB [30,31]. Therefore, we hypothesized that BDNF and NGF were the NFκB-regulated functional factors mediating SCF+G-CSF-induced enhancement of neurite outgrowth. To test this hypothesis, we first examined whether SCF and G-CSF have the ability to induce upregulation of both BDNF and NGF gene expression through the regulation of NFκB. Using the approach of qRT-PCR, we quantified the gene expression of BDNF and NGF at 6 and 24h after treatment with the medium, SCF, G-CSF, SCF+G-CSF, SCF+NFκB inhibitor, G-CSF+NFκB inhibitor or SCF+G-CSF+NFκB inhibitor. SCF and G-CSF alone or in combination treatment were performed 24h after seeding, and NFκB inhibitor was added into the neuron culture medium 1h before treatment. The data of BDNF gene expression showed that SCF alone or in combination with G-CSF (SCF+G-CSF) caused significant increases in BDNF gene expression at both 6 and 24h after treatment as compared to medium controls (p <0.01) (Figure 7A). Additionally, SCF- or SCF+G-CSF-induced upregulation of BDNF gene expression was significantly blocked by BAY 11-7082, the inhibitor for NFκB, at both time points (p <0.01) (Figure 7A). The G-CSF alone treatment only induced an elevation of BDNF gene expression at 24h but not at 6h after treatment, and the G-CSF-induced upregulation of BDNF gene expression at 24 h was also significantly inhibited by BAY 11-7082 (p <0.01) (Figure 7A). Notably, SCF+G-CSF-induced BDNF gene expression was significantly higher than those of SCF or G-CSF alone treatment at both 6 and 24h after treatment, suggesting synergistic and adjuvant effects of SCF+G-CSF on BDNF gene expression (p <0.01) (Figure 7A). When we analyzed the NGF gene expression data, we observed that SCF or SCF+G-CSF significantly increased NGF gene expression at both 6 and 24h after treatment in comparison with medium controls or G-CSF alone treatment, and that BAY 11-7082 significantly prevented SCF- or SCF+G-CSF-induced upregulation of NGF gene expression at both time points (p <0.01) (Figure 7B). Unlike BDNF gene regulation, SCF+G-CSF did not demonstrate synergistic effects to increase NGF gene expression. G-CSF alone did not display any upregulative effects on NGF (Figure 7B). These data indicate that SCF and SCF+G-CSF can enhance both BDNF and NGF gene expression through the regulation of NFκB.

To further identify whether SCF+G-CSF-induced upregulation of BDNF and NGF gene expression is specific, siRNAs against BDNF or NGF was used to silence the genes of BDNF and NGF, respectively. Knockdown of BDNF and NGF by siRNAs was examined at the levels of mRNA and protein. Peptide RVG-9R was used to deliver siRNAs to the neurons because it has been shown that this peptide is effective in binding and specifically transducing siRNAs into the neurons [32]. Twenty-four hours after seeding, negative control siRNAs or the siRNAs against BDNF or NGF were introduced to the neurons. One hour after adding siRNAs, cortical neurons were treated with or without SCF and G-CSF. In the first experiment, BDNF and NGF gene expression were examined with qRT-PCR 36h after exposure to siRNAs. We chose this time point for determination of BDNF and NGF gene silencing because the peak of silencing mRNAs by siRNAs appears between 24-48h after introducing siRNAs according to the manufacturer’s instruction (Applied Biosystems). In line with the findings mentioned earlier (Figure 7 A and B), we found that the level of BDNF mRNAs was significantly elevated by SCF+G-CSF or SCF+G-CSF+negative control siRNAs as compared to medium controls (p < 0.01, Figure 7C). In addition, SCF+G-CSF-induced upregulation of BDNF mRNAs was significantly blocked by anti-BDNF siRNAs (p < 0.01, Figure 7C). When analyzing NGF mRNA data, we observed the same results as obtained in the BDNF gene expression study that both the SCF+G-CSF and SCF+G-CSF+negative control siRNAs showed significant increases in NGF mRNAs (p < 0.01, Figure 7D). The NGF siRNAs significantly inhibited SCF+G-CSF-induced upregulation of NGF mRNA expression (p < 0.01, Figure 7D).

In the second experiment, we examined the levels of BDNF and NGF proteins with Western-Blotting 3 days after adding siRNAs. We selected day 3 to test the protein levels because the target protein can be effectively blocked 3 days after introducing siRNAs according to the manufacturer’s instruction (Applied Biosystems). We found that BDNF protein was significantly increased by SCF+G-CSF and SCF+G-CSF+negative control siRNAs as compared to medium controls (p < 0.05) (Figure 7 E and G). The SCF+G-CSF-induced the increase in BDNF protein was significantly blocked by anti-BDNF siRNAs (p < 0.05) (Figure 7 E and G). SCF alone also showed a significant increase in BDNF protein in comparison with medium controls (p < 0.05, Figure 7 E and G), whereas G-CSF alone only showed a trend toward increased BDNF protein, but it did not reach a significant level (p > 0.05, Figure 7 E and G). Similar to the BDNF data, SCF+G-CSF also caused a significant increase in NGF protein production, and the SCF+G-CSF-induced increase of NGF was significantly prevented by anti-NGF siRNAs (p < 0.05, Figure 7 F and H). SCF displayed a trend toward increased NGF, whereas the level of NGF in G-CSF treatment showed no difference from the medium controls (Figure 7 F and H).

Together, these observations indicate that SCF+G-CSF-induced upregulation of BDNF and NGF at both the mRNA level and protein level is specific.

**Figure 8:**
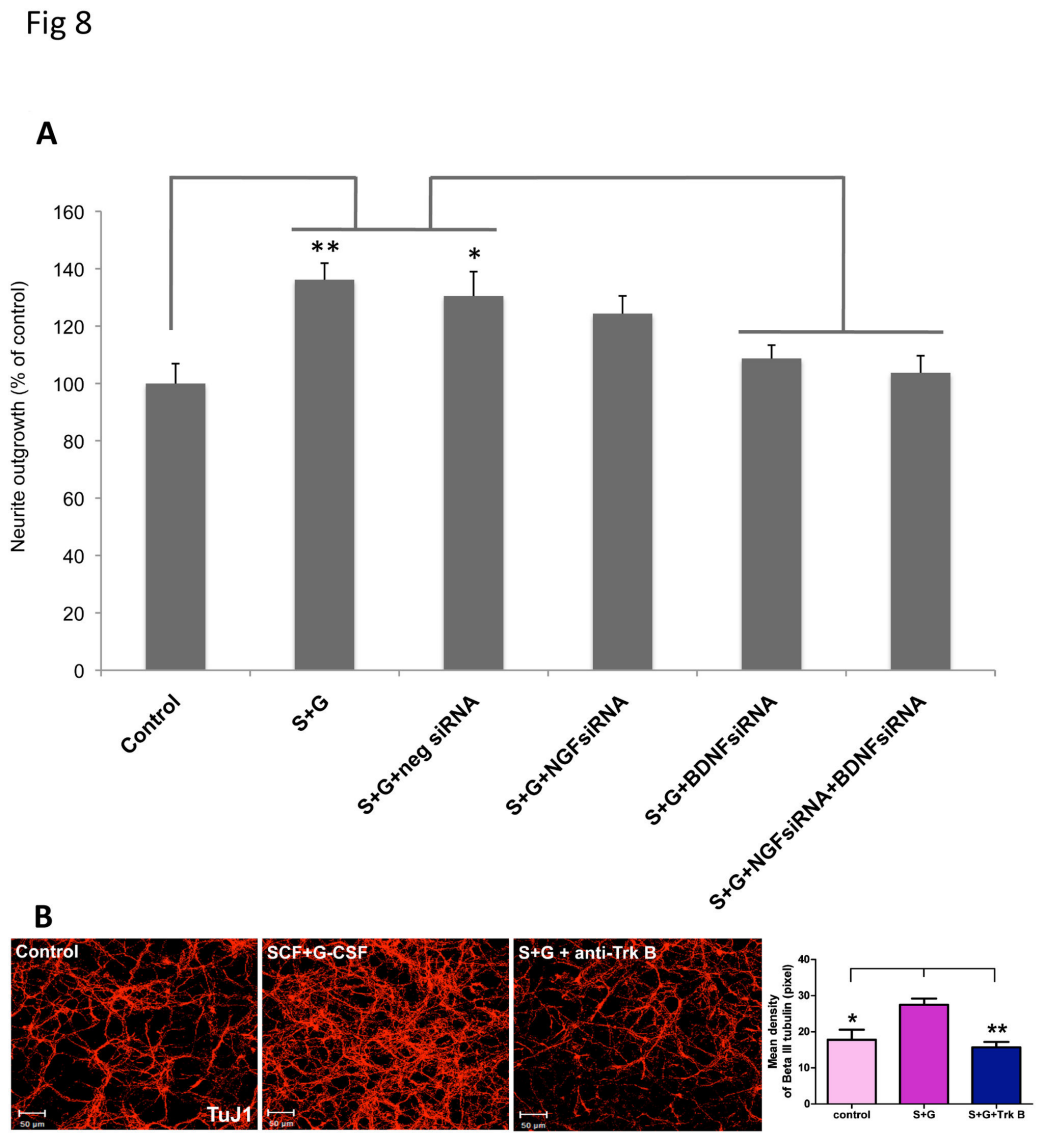
The involvement of BDNF in SCF+G-CSF-induced enhancement of neurite outgrowth. (**A**) The role of BDNF and NGF on SCF+G-CSF-induced enhancement of neurite outgrowth. Neurite outgrowth was quantified with a Neurite Outgrowth Quantification kit 72h after introducing siRNAs to neurons. SCF and G-CSF were added to neurons one hour after siRNA transfection. Note that SCF+G-CSF treatment results in a significant enhancement of neurite outgrowth. In addition, SCF+G-CSF-induced neurite outgrowth is significantly blocked by BDNF siRNA or BDNF siRNA+NGF siRNA, whereas BDNF siRNA+NGF siRNA treatment has no significant difference as compared to the BDNF siRNA treatment. NGF siRNA shows a trend towards decreasing SCF+G-CSF-induced neurite outgrowth. Negative siRNAs do not affect the SCF+G-CSF-induced the enhancement of neurite outgrowth as the neurite outgrowth in the negative siRNA-treated neurons still remains significantly greater than those of medium controls and BDNF siRNA+NGF siRNA. Neg: negative controls for siRNA. **p < 0.01, *p < 0.05. Mean ± SE, n=8. (**B**) SCF+G-CSF-induced enhancement of neurite outgrowth is mediated by BDNF receptor, TrkB. The neurite outgrowth was tested 72 h after treatment. Note that SCF+G-CSF-induced the increase in neurite extension (SCF+G-CSF vs. control, *p < 0.05) is significantly blocked by an anti-TrkB antibody (SCF+G-CSF vs. SCF+G-CSF+TrkB antibody, **p < 0.01). Scale bars, 50µm. Mean ± SE, n=3.

Next we then determined whether BDNF and NGF were both critically involved in SCF+G-CSF-induced enhancement of neurite outgrowth. As mentioned earlier, we obtained the evidence that BDNFsiRNA and NGFsiRNA blocked SCF+G-CSF-induced elevation of BDNF and NGF, respectively (Figure 7). In this experiment we used the siRNAs against BDNF and NGF to examine the role of BDNF and NGF in the SCF+G-CSF-induced enhancement of neurite outgrowth. The siRNAs, SCF and G-CSF were added into the cortical neurons in the same manner as stated earlier in the siRNA experiment. To examine and quantify neurite outgrowth, in this experiment neurons were grown on the transwell membranes, and neurite outgrowth was examined with a Neurite Outgrowth quantification kit 72h after treatment. The neurite outgrowth data showed that SCF+G-CSF caused a significant increase in neurite outgrowth (P < 0.01 vs. control) (Figure 8 A), which is in line with the findings mentioned earlier (Figures 2 and 3). In addition, administration of anti-BDNF siRNA + anti-NGF siRNA significantly prevented SCF+G-CSF-induced neurite outgrowth (P < 0.01, Figure 8 A). Anti-BDNF siRNA alone also showed a significant reduction in SCF+G-CSF-induced neurite outgrowth (P < 0.01, Figure 8 A) whereas anti-NGF siRNA alone only showed a trend toward decreasing SCF+G-CSF-induced neurite outgrowth but it did not reach the significant level (Figure 8 A). In addition, anti-BDNF siRNA + anti-NGF siRNA did not further decrease SCF+G-CSF-induced neurite outgrowth as compared to anti-BDNF siRNA alone, suggesting that anti-NGF siRNA has less effect than anti-BDNF siRNA to prevent SCF+G-CSF-induced neurite outgrowth. Negative siRNA controls did not block the effect of SCF+G-CSF on neurite outgrowth because the neurite outgrowth in this treatment was still greater than those of the medium controls and the SCF+G-CSF+BDNFsiRNA+NGFsiRNA treatment (P < 0.05, Figure 8 A). Taken together, these data suggest that BDNF is the mediator for SCF+G-CSF-induced enhancement of neurite outgrowth.

Lastly, we examined the contribution of released BDNF in SCF+G-CSF-induced enhancement of neurite outgrowth. BDNF has been shown to promote neuronal process extension in autocrine [33] and paracrine manners [34]. In this experiment, antibody against TrkB, the receptor for BDNF, was added into the neuron medium 1h before SCF+G-CSF treatment, and the treatment started at 24h after seeding neurons. Neurite outgrowth was determined 72h after plating. As shown in Figure 8 B, SCF+G-CSF-induced enhancement of neurite outgrowth was significantly blocked by the anti-TrkB antibody, suggesting that SCF+G-CSF may enhance neurite extension through the autocrine and paracrine actions of BDNF.

## Discussion

Regulation of HSC/HPC survival, proliferation and differentiation was discovered as the effects of SCF and G-CSF [5,6], and SCF+G-CSF has a synergistic effect on the mobilization of HSCs/HPCs [7]. Here we have demonstrated a novel role for SCF+G-CSF in synergistic regulation of neurite extension. Our data show that receptors for SCF and G-CSF are expressed in the cortical neurons including the growth cores of neurites. SCF+G-CSF displays greater effects to enhance neurite outgrowth and the neurite outgrowth-related signaling as compared to SCF or G-CSF alone. SCF shows similar effects as SCF+G-CSF; however, the efficiency of SCF is much less than SCF+G-CSF. Although G-CSF causes a short duration of NFκB activation and slightly upregulates BDNF gene expression in a delayed manner, a significant increase in neurite outgrowth is not induced by G-CSF. Interestingly, these data are in agreement with our previous study of chronic stroke using a rat model of cerebral cortical ischemia. We found that only SCF+G-CSF treatment induced a stable and long-term functional improvement. SCF displayed an unstable functional restoration, whereas G-CSF did not show a functional benefit [16]. In a live brain imaging study, we also observed that SCF+G-CSF treatment in chronic stroke enhanced neuronal network remodeling in the peri-infarct cortex [19]. Together, these data suggest that hematopoietic growth factors, SCF+G-CSF, have ectopic effects on neurite extension and neuronal network rewiring in addition to their effects on HSCs/HPCs.

PI3K/AKT signaling is involved in SCF+G-CSF-induced enhancement of neurite outgrowth. PI3K/AKT pathway has been shown to play an important role in the regulation of neurite growth. Microinjection of activated PI3K [35] or the expression of a constitutively active PI3K [36] in PC12 cells promotes neurite growth. It has been demonstrated that growth factor-induced neurite extension is also mediated by PI3K/AKT signaling. Blocking of the PI3K/AKT pathway inhibits the neurite outgrowth induced by NGF [37], BDNF [21], or insulin-like growth factor-1 [38]. Our current research provides additional evidence showing the involvement of PI3K/AKT signaling in SCF+G-CSF-induced enhancement of neurite outgrowth.

NFκB activation and transcriptional regulation are synergistically enhanced by SCF+G-CSF. As mentioned earlier, NFκB is retained inactive in the cytoplasm by interaction with IκB family of inhibitory proteins. In neurons, the most common NFκB complex in the cytoplasm consists of p50/p65 and IκBα [39]. Phosphorylation of IκBα on serine residues 32 and 36, or tyrosine 42 [24,40] causes NFκB activation. The freed NFκB (p50/p65) translocates into the nucleus, binds to κB consensus sequence in the promoter or enhancer regions of target genes, and activates their transcription. Here we have revealed that SCF and G-CSF alone or in combination cause rapid increases in IκBα phosphorylation of cortical neurons 15 and 30 min after treatment. Additionally, the level of phosphorylated IκBα is synergistically elevated by SCF+G-CSF as compared to SCF or G-CSF alone. It remains to be determined, however, how SCF+G-CSF induces the synergistic effect on IκBα phosphorylation. Interestingly, Duarte and Frank [7] reported that SCF+G-CSF synergistically enhances myeloid cell proliferation through the synergistic regulation of transcription factor (STAT3) and proliferation-related gene (c-fos) expression, and that the synergistic activation of STAT3 by SCF+G-CSF is coordinated with SCF-induced phosphorylation of STAT3 on serine727 and G-CSF-caused tyrosine phosphorylation of STAT3. Using the phospho-IκBα ^Ser32/Ser36^ ELISA kit, our data show that SCF+G-CSF induces synergistic effects on IκBα phosphorylation in serine residues 32 and 36. How SCF+G-CSF induces such a synergistic effect on the phosphorylation of IκBα in the serine residues 32 and 36 is the open question to be addressed in future studies. In addition to the synergistic effects of SCF+G-CSF on IκBα phosphorylation, SCF+G-CSF also shows synergistic effects on the regulation of NFκB transcriptional activity. As mentioned earlier, a rapid increase in IκBα phosphorylation is seen 15 and 30 min after SCF, G-CSF, or SCF+G-CSF treatment. Interestingly, only SCF+G-CSF induces greater and long-lasting NFκB binding that is seen 3 and 6 hours after treatment as compared to SCF and G-CSF alone. Although it is not clear how SCF+G-CSF enhances the NFκB transcriptional binding in such a long duration period, previous studies have shown that NFκB (p65/p50) binding to the κB site on DNA is required for adjacent enhancer elements and p65-recuited co-activators [23,39]. Moreover, NFκB promotes IκBα gene expression, re-synthesized IκBα can mask the DNA-bound dimmers of NFκB, and the masked NFκB is exported out to the cytoplasm [41,42]. Therefore, the SCF+G-CSF-induced long-lasting effects on NFκB transcriptional activity may be orchestrated by SCF+G-CSF through the positive regulation of the co-activators and/or enhancers and the negative regulation of IκBα activation and/or production.

SCF+G-CSF-induced enhancement of neurite outgrowth is dependent on long-term NFκB activation. NFκB signaling is crucially involved in neuron survival, neuronal process formation, synaptic plasticity and learning and memory [23]. Many studies have documented that NFκB is required for neurite outgrowth. Inhibiting NFκB activation with an IκBα phosphorylation inhibitor or proteosomal degradation inhibitor, or inhibiting NFκB transcriptional activity with κB decoy DNA leads to a reduction in the size and complexity of neurite arbors in cultured neurons [43]. Further, ciliary neurotrophic factor [40] or NGF [44] enhances neurite outgrowth depending on NFκB. It is worth noting that NFκB promotes neuron survival when activated over a long time period [41,45,46]. Is prolonged activity of NFκB transcription also critical for neurite outgrowth? Our data show that SCF+G-CSF induces a long-lasting increase in NFκB transcriptional activity (1-6 hours post-treatment), and that SCF+G-CSF-induced neurite outgrowth is dependent on NFκB activity. By contrast, G-CSF alone does not induce NFκB transcriptional activity longer than 1h nor dose it increase neurite outgrowth. Although SCF also increases NFκB binding at 1 and 3 hours post-treatment, the level of NFκB binding is significantly lower than SCF+G-CSF at 3 hours post-treatment. The SCF-induced an increase in neurite outgrowth is also shown to be much less than SCF+G-CSF. These data suggest that a long-lasting increase in NFκB transcriptional activity is necessary for the enhancement of neurite outgrowth by SCF+G-CSF.

SCF+G-CSF increases both BDNF and NGF through the regulation of NFκB, and BDNF, but not NGF, is involved in the SCF+G-CSF-induced enhancement of neurite outgrowth in cortical neurons. NGF and BDNF belong to the neurotrophin family and bind their receptors, tropomyosin-receptor-kinase A (TrkA) or TrkB, respectively, to govern neuronal differentiation, survival, neurite outgrowth and synaptic functions [47] through an autocrine and/or paracrine fashion. Both NGF [30] and BDNF [31] gene expression is regulated by NFκB. Our data show that NFκB is the key mediator for the upregulation of NGF and BDNF by SCF+G-CSF. However, only BDNF, but not NGF, is required for SCF+G-CSF-induced neurite growth in cortical neurons. Other investigators have noted the similar phenomenon that BDNF, but not NGF, promotes neurite outgrowth in retinal neurons [48], vestibulospinal neurons [49], and pontocerebellar mossy fiber neurons [47]. This diversity in the effect of NGF and BDNF on neurite outgrowth may be related to the distribution of their receptors. BDNF and TrkB have a widespread distribution in the CNS [50] and they are highly expressed in the cortex [51]. By contrast, TrkA is expressed at very low levels in the cortex [52]. These findings may help in understanding why both BDNF and NGF are increased by SCF+G-CSF, while only BDNF is critically involved in the SCF+G-CSF-induced enhancement of neurite growth in the cortical neurons. It has been demonstrated that BDNF stimulates neuronal process outgrowth in both autocrine [32] and paracrine manners [34]. Our data show that an anti-TrkB antibody completely prevents the effects of SCF+G-CSF on neurite growth in the cortical neurons. This observation suggests that SCF+G-CSF-upregulated BDNF may act in an autocrine and/or paracrine fashion via its TrkB receptor to regulate neurite outgrowth of cortical neurons.

In summary, we have revealed that SCF+G-CSF can directly and synergistically enhance neurite outgrowth through PI3K/AKT and NFκB/BDNF signaling. These data provide new insights into the contribution of the hematopoietic growth factors, SCF and G-CSF, in neuronal plasticity. The outgrowth of neural processes is essential for the establishment of neuronal networks in the CNS during development and brain repair, and it is also a critical feature of neural plasticity throughout the lifespan. In addition to the data collected from perinatal cortical neurons in vitro, our recent study has showed that SCF+G-CSF also increases axonal branching through NFkB pathway in adult brain in an animal model of chronic stroke (Cui et al., unpublished observation). Thus, our findings would help in understanding the role of hematopoietic growth factors in CNS development, brain plasticity, and brain repair in the setting of brain injury or disease.

## Supporting Information

Movie S1 **Dynamics of neurite extension in medium only (medium control) during 8-26 h after seeding cortical neurons.** Note that the direction of neurite outgrowth is changing dynamically over time and that neurite extension and retraction also occurs during the recording period.
(MP4)

Movie S2 **Dynamics of neurite extension in SCF+G-CSF treatment during 8-26 h after seeding cortical neurons.** Note that the SCF+G-CSF-treated neuron displays polarization and neurite outgrowth much more quickly after treatment as compared to the medium control. Additionally, fine neurites with multiple branches are generated by SCF+G-SCF treatment when compared to the medium control.
(MP4)

Movie S3 **Dynamics of neurite extension in SCF+G-CSF+NFkB inhibitor treatment during 8-26 h after seeding cortical neurons.** An NFκB inhibitor, BAY 11-7082 (10µM), was added 60 min prior to SCF+G-SCF treatment. Note that SCF+G-CSF-induced enhancement of neurite outgrowth and branching is dramatically prevented.
(MP4)
